# Social media use, habits and attitudes toward e-professionalism among medicine and dental medicine students: a quantitative cross-sectional study

**DOI:** 10.3325/cmj.2021.62.569

**Published:** 2021-12

**Authors:** Joško Viskić, Dražen Jokić, Marko Marelić, Lovela Machala Poplašen, Danko Relić, Kristijan Sedak, Tea Vukušić Rukavina

**Affiliations:** 1 Department of Fixed Prosthodontics, School of Dental Medicine, University of Zagreb, Zagreb, Croatia; 2 Department of Orthodontics, School of Dental Medicine, University of Zagreb, Zagreb, Croatia; 3 Department of Medical Sociology and Health Economics, Andrija Stampar School of Public Health, University of Zagreb, School of Medicine, Zagreb, Croatia; 4 Andrija Stampar Library, Andrija Stampar School of Public Health, School of Medicine, University of Zagreb, Zagreb, Croatia; 5 Department of Medical Statistics, Epidemiology and Medical Informatics, Andrija Stampar School of Public Health, University of Zagreb, School of Medicine, Zagreb, Croatia; 6 Center for Career Planning in Biomedicine and Health, University of Zagreb, School of Medicine, Zagreb, Croatia; 7 Department of Communication Sciences, Catholic University of Croatia, Zagreb, Croatia

## Abstract

**Aim:**

To describe and compare social media (SM) use and habits, and attitudes of medical and dental students toward e-professionalism and to determine their opinion on potentially unprofessional behavior and posts.

**Methods:**

In this quantitative cross-sectional questionnaire study, students of the University of Zagreb School of Medicine and those of the School of Dental Medicine completed a survey-specific questionnaire on the use of SM, SM habits, and attitudes toward e-professionalism.

**Results:**

Of the 714 collected questionnaires, we analyzed 698 (411 from medical and 287 from dental students). The most commonly used SM were Facebook (99%) and Instagram (80.7%). Unprofessional content was recognized by both groups. Medical students significantly more frequently considered the posts containing patient photos (61% vs 89.8%; *P* < 0.001), describing interaction with a patient not revealing any personal identifiable information (23% vs 41.8%; *P* < 0.001), and containing critical comments about faculty (53% vs 39.7%; *P* = 0.001) to be unprofessional. Dental medicine students were significantly more open to communication through SM (39.7% vs 16.3%; *P* < 0.001), more often reported that they would accept (41.5% vs 12.2%; *P* < 0.001), and had accepted (28.2% vs 5.6%; *P* < 0.001) friend requests/follows/tracks from patients, and sent friend requests/follows/tracks to their patients (5.2% vs 1.2%; *P* = 0.002).

**Conclusion:**

Both groups were highly aware of e-professionalism. Dental students were more desensitized to visual representations of patients, and more prone to SM interactions with patients, which might expose them to the risk of unprofessional behavior.

Social media (SM) use has increased among health professionals of all levels (1). Kaplan et al defined SM as “various forms of media content that are publicly available and created by end-users” (2). On SM, users post content and interact frequently and abundantly. Individuals generally use SM to cultivate interpersonal relationships, to establish and promote a real or semi-real self-image, and to access entertaining and educational content (3). The student population is at the forefront of this technological trend and has been immersed in this media form for the majority of their lives.

Professional behavior online is paramount for health professionals as violating the strict ethical and legal boundaries may result in board disciplinary proceedings, monetary fines, and even license restrictions or suspensions (4,5). The term “e-professionalism” was defined as “attitudes and behaviors (some of which may occur in private settings) reflecting traditional professionalism paradigms that are manifested through digital media” (6).

Patients are also increasingly active online, searching the internet for health-related information and information about health providers (7). As online activity is digitally recorded and stored, the “digital footprint” is impossible to erase (8). With both the student and patient population being present on SM, interaction is inevitable. Although students are not yet health professionals, they should obey the same ethical and professional rules as health professionals do. This combination of factors presents a possible concern because of the unclear boundaries of SM interaction and uncontrolled audience. It is necessary to understand students’ use of SM, as well as their SM habits and attitudes toward SM. The student population has a higher prevalence of worrying use of SM sites, and the problem was linked with lower scores of subjective well-being (9). Health care educators also expressed interest and worry in these matters (10,11).

Although medicine and dental medicine are similar professions, they differ in some aspects. Dentistry has become more business-driven, and practitioners have to balance between clinical and commercial motives (12). This can affect the students' motivation to choose one of these professions, which can also lead to different habits and attitudes of medical and dental students on SM (13,14).

Both medical and dental students are aware of the standards online professional behavior, but serious transgressions (postings of alcohol abuse, drug use, negative posts regarding persons gender/race/disability, etc) have been identified in both groups (15,16). No studies so far directly compared these two student populations. As these two professions collaborate in joined private and public practices, and will interact with peers, superiors, and patients on SM in the future, it is important to understand the similarities and differences in their SM habits and attitudes on professional behavior on SM. In addition, it is essential to create guidelines and form curriculum-integrated educational content that suits their views and understanding of e-professionalism. The aims of this study were to describe and compare SM use and habits by medical and dental students. We also compared their attitudes on e-professionalism and assessed their opinion on potentially unprofessional behavior on SM.

## Participants and methods

### Design

This quantitative cross-sectional study was conducted at the University of Zagreb School of Medicine (UZSM) and the University of Zagreb School of Dental Medicine (UZSDM) in the academic year 2018/2019.

### Study instrument

Data were collected by using a survey-specific questionnaire named “Exploring the Impact of Social Networks on the Professional Behavior of Healthcare Professionals” developed on the basis of the literature search (17-20). The questionnaire was composed of seven instruments that measured 1) sociodemographic characteristics and habits of SM usage; 2) knowledge of SM; 3) reasons of SM usage; 4) impression management on SM; 5) security on SM; 6) attitude toward professionalism; and 7) attitude toward e-professionalism (Supplementary material(Supplementary material)).

None of the previously used instruments or scales comprehensively covered the seven domains of our interest for this study. Therefore, we integrated into our questionnaire the most beneficial parts of the existing questionnaires and added specific areas of interest that were not covered previously. The questionnaire was internally reviewed for content validity by a multi-disciplinary expert team in the field (psychiatrist, sociologist, communicologist, doctor of medicine, and doctor of dental medicine) (21,22) to theoretically assess whether these domains adequately measured our research objectives (23,24). The final version was created and made available online by means of survey-generating application Google Forms. The first questionnaire item refers to providing informed consent (with the “opt out” option) and is followed by 43 questions. The study and the questionnaire were approved by the Ethics Committees of UZSM (641-01/18-02/01) and UZSDM (05-PA-24-2/2018).

### Data collection and analysis

The questionnaire was available on the official medical and dental school project website from October 2018 to April 2019. During regular classes, students were informed about the possibility to complete the questionnaire (second- and fifth-year UZSM students and UZSDM students of all 6 years). The participation was voluntary, and no incentive was given for survey completion. To ensure anonymity, identification information was not collected. By default, the data were not collected in Google Forms if the questionnaire was not completed. Duplicated data were excluded. If the respondents indicated they were not SM users, their negative response was collected, they were redirected to the end of the questionnaire, and their responses were not included in the analysis.

### Statistical analysis

Demographic data are presented as descriptive statistics. Medical and dental students' responses were compared with the χ^2^ or Fisher exact test in the cases when more than 20% of cells had expected value equal or lower than five. The significance level was set at a two-tailed alpha of 0.05. Statistical analysis was conducted in SPSS, version 25.0 (IBM Corp., Armonk, NY, USA).

## Results

Of 714 questionnaires collected, 16 were excluded (1 respondent did not give informed consent; 4 stated that they were younger than 10 years; 2 questionnaires were identical duplicates that occurred before the internet connection was disconnected during responding; 9 respondents indicated no use of SM). A total of 698 respondents were included in the analysis: 411 second- and fifth-year UZSM students (response rate 69%) and 287 first- to sixth-year UZSDM students (response rate 49.7%). The sample was predominantly female (73.9%), with a median age of 22. This article presents the results of two out of the seven instruments: 1) socio-demographic characteristics and habits of SM use; 7) attitudes toward e-professionalism. Demographical data are shown in Table 1.

**Table 1:** Distribution of students by sex, age, and the study year

	All students n (%)	Medical n (%)	Dental n (%)
**Sex**			
male	182 (26.1)	137 (33.3)	45 (15.7)
female	516 (73.9)	274 (66.7)	242 (84.3)
total	698 (100)	411 (100)	287 (100)
**Age**			
average	21.71	21.6	21.86
median	22	22	22
min	18	18	18
max	30	30	30
standard deviation	1.985	1.963	2.008
**Study year**			
first year	44 (6.3)	0	44 (15.3)
second year	243 (34.8)	204 (49.6)	39 (13.6)
third year	43 (6.2)	0	43 (15.0)
fourth year	60 (8.6)	0	60 (20.9)
fifth year	265 (37.9)	207 (50.4)	58 (20.2)
sixth year	43 (6.2)	0	43 (15.0)
total	698 (100)	411 (100)	287 (100)

The questionnaire was used to assess the forms and type of SM use, as well as the access frequency and device type (Table 2). Medical students significantly more frequently used YouTube (76.9% vs 65.2%; *P* = 0.001).

**Table 2:** Social media usage, habits, and device preference

	All students n (%)	Medical n (%)	Dental n (%)	χ^2^, df, *P*
**Social media platforms**				
Facebook	691 (99.0)	405 (98.5)	286 (99.7)	2.103, 1, 0.250
Instagram	563 (80.7)	322(78.3)	241 (84.0)	3.430, 1, 0064
Pinterest	193 (27.7)	109 (26.5)	84 (29.3)	0.638, 1, 0.425
Tumblr	88 (12.6)	56 (13.6)	32 (11.1)	0.940, 1, 0.332
Twitter	117 (16.8)	74 (18.0)	43 (15.0)	1.106, 1, 0.293
LinkedIn	63 (9.0)	44 (10.7)	19 (6.6)	3.435, 1, 0.064
YouTube	503 (72.1)	316 (76.9)	187 (65.2)	11.547, 1, 0.001
Google+	386 (55.3)	225 (54.7)	161 (56.1)	0.125, 1, 0.724
**Active/passive usage**				
more active than passive	42 (6.0)	25 (6.1)	17 (5.9)	1.854, 2, 0.396
half-and-half	209 (29.9)	115 (28.0)	94 (32.8)	
more passive than active	447 (64.0)	271 (65.9)	176 (61.3)	
**Access frequency**				
More than 10 × a day	227 (32.5)	134 (32.6)	93 (32.4)	9.447, 4, 0.051
5 to 10 × a day	236 (33.8)	143 (34.8)	93 (32.4)	
2 to 4 × a day	152 (21.8)	76 (18.5)	76 (26.5)	
once a day	35 (5.0)	25 (6.1)	10 (3.5)	
2 × to 3 × a week or less	48 (6.9)	33 (8.0)	15 (5.2)	
**Device**				
desktop computer	7 (1.0)	7 (1.7)	0 (0.0)	4.942, 2, 0.084
laptop computer	5 (0.7)	3 (0.7)	2 (0.7)	
mobile device	686 (98.3)	401 (97.6)	285 (99.3)	
**Purpose of use**				
exclusively personal	284 (40.7)	159 (38.7)	125 (43.6)	2.930, 2, 0.231
personal and professional	412 (59.0)	250 (60.8)	162 (56.4)	
exclusively professional	2 (0.3)	2 (0.5)	0 (0.0)	

Medical and dental students’ responses regarding what behaviors/posts they consider unprofessional on SM are shown in Table 3. Most respondents believed that the posts containing the following content were unprofessional: patient information (93.9% medical vs 90.9% dental), drug use (91.3% dental vs 91% medical), and overtly sexual content (92% dental vs 87.3% medical). Medical students significantly more frequently considered the following types of posts as unprofessional: posts containing patients' photos (89.8% vs 61%; *P* < 0.001); those describing interaction with a patient not revealing any personal identifiable information (41.8% vs 23%; *P* < 0.001); and those advertising pharmaceutical or health products without disclosing any conflict of interest (41.1% vs 26.8%; *P* < 0.001). Dental medicine students significantly more frequently considered the following types of posts as unprofessional: posts with swearing or inappropriate language (83.3% vs 72%; *P* = 0.001); those showing obscene gestures in photos (75.6% vs 65.2%; *P* = 0.003); those showing partial nudity (71.1% vs 63%; *P* = 0.027); those displaying critical comments about faculty (53% vs 39.7%; *P* = 0.001); those stating opinions on controversial issues (38% vs 27.5%; *P* = 0.003); those displaying the membership of certain online groups dealing with controversial issues (38.3% vs 27.3%; *P* = 0.002;), and those displaying critical comments about teaching materials, study program, school, or university (36.2% vs 25.1%; *P* = 0.001).

**Table 3:** Students' opinions about potentially unprofessional behavior and posts on social media (SM)

Which of the following types of posts/behaviors (posted on SM) do you consider unprofessional?				
	all students n (%)	medical n (%)	dental n (%)	χ^2^, df, *P*
Posts that display patient information	647 (92.7)	386 (93.9)	261 (90.9)	2.211, 1, 0.137
Posts that show illicit drug use	636 (91.1)	374 (91.0)	262 (91.3)	0.018, 1, 0.894
Photos of patients	544 (77.9)	369 (89.8)	175 (61.0)	81.547, 1, <0.001
Posts showing misdemeanors	618 (88.5)	364 (88.6)	254 (83.5)	0.001, 1, 0.980
Posts that include overtly sexual content	623 (89.3)	359 (87.3)	264 (92.0)	3.791, 1, 0.052
Images of a person who looks undeniably drunk	520 (74.5)	306 (74.5)	214 (74.6)	0.010, 1, 0.973
Swearing or inappropriate language	535 (76.6)	296 (72.0)	239 (83.3)	11.962, 1, 0.001
Posting a status update to describe considerable alcohol consumption at a party	465 (66.6)	269 (65.5)	196 (68.3)	0.614, 1, 0.433
Obscene gestures in photos (middle finger, etc.)	485 (69.5)	268 (65.2)	217 (75.6)	8.625, 1, 0.003
Posts that contain partial nudity	463 (66.3)	259 (63.0)	204 (71.1)	4.920, 1, 0.027
Expressing attitudes of superiority (based on professional status)	414 (59.3)	251 (61.1)	163 (56.8)	1.280, 1, 0.258
Posts that describe interaction with a patient, not revealing any personal identifiable information	238 (34.1)	172 (41.8)	66 (23.0)	26.729, 1, <0.001
Advertising of pharmaceutical or health products without disclosing any conflict of interest	246 (35.2)	169 (41.1)	77 (26.8)	15.121, 1, <0.001
Critical comments about faculty	315 (45.1)	163 (39.7)	152 (53.0)	12.076, 1, 0.001
Image of a person drinking alcohol	195 (74.5)	114 (27.7)	81 (28.2)	0.020, 1, 0.888
Stating opinions in comments on controversial issues	222 (31.8)	113 (27.5)	109 (38.0)	8.566, 1, 0.003
Display of the membership of certain online groups dealing with controversial issues	222 (31.8)	112 (27.3)	110 (38.3)	9.560, 1, 0.002
Critical comments about teaching materials, study program, school, or university	207 (29.7)	103 (25.1)	104 (36.2)	10.118, 1, 0.001
Display of the current love status	67 (9.6)	39 (9.5)	28 (9.8)	0.014, 1, 0.906

Students’ opinions about online professionalism standards are displayed in Table 4. Four-point scale was recoded as a binary variable to clarify responses. “Strongly disagree” and “disagree” was recoded as “disagree” and “agree” and “strongly agree” as “agree”. Only 22.3% of the respondents believed that it was always possible to maintain professionalism in online activities, and 64.2% believed that their online activities did not affect them as professionals. Just a fraction fewer (63.9%) believed they should be allowed to do whatever they wanted online. Dental students were significantly more prone to such views (68.3% vs 60.8%, *P* = 0.043). The majority (80.1%) rejected the idea of the school controlling their online activity, and disagreed with limiting (78.9%) or completely banning (87.7%) SM use for health professionals.

**Table 4:** Students' opinions about professionalism standards related to social media activities

	All students n (%)	Medical n (%)	Dental n (%)	χ2, df, P
Professionalism in online activities is as important as in traditional (offline) environments.				
Disagree	116 (16.6)	76 (18.5)	40 (13.9)	2.529, 1, 0.112
Agree	582 (83.4)	335 (81.5)	247 (86.1)	
It is not always possible to maintain professionalism in online activities.				
Disagree	156 (22.3)	90 (21.9)	66 (23)	0.118, 1, 0.732
Agree	542 (77.7)	321 (78.1)	221 (77)	
People have the opportunity to post photos and document aspects of their professional life that would otherwise remain private.				
Disagree	95 (13.6)	65 (15.8)	30 (10.5)	4.132, 1, 0.026
Agree	603 (86.4)	346 (84.2)	257 (89.5)	
Social media have removed protection of professionals against the public.				
Disagree	258 (37)	148 (36)	110 (38.3)	0.390, 1, 0.293
Agree	440 (63)	263 (64)	177 (61.7)	
Professionals cannot actually fully relax.				
Disagree	236 (33.8)	132 (32.1)	104 (36.2)	1.282, 1, 0.258
Agree	462 (66.2)	279 (67.9)	183 (63.8)	
The risks of social networking software greatly overweigh the benefits.				
Disagree	416 (59.6)	246 (59.9)	170 (59.2)	0.027, 1, 0.869
Agree	282 (40.4)	165 (40.1)	117 (40.8)	
Healthcare professionals should be restricted from using social networking software due to too much of a risk.				
Disagree	551 (78.9)	328 (79.8)	223 (77.7)	0.450, 1, 0.502
Agree	147 (21.1)	83 (20.2)	64 (22.3)	
Healthcare professionals should be banned from using social networking software due to too much of a risk.				
Disagree	612 (87.7)	363 (88.3)	249 (86.8)	0.381, 1, 0.537
Agree	86 (12.3)	48 (11.7)	38 (13.2)	
I believe that my online activities do not affect me as a professional.				
Disagree	250 (35.8)	140 (34.1)	110 (38.3)	1.337, 1, 0.248
Agree	448 (64.2)	271 (65.9)	177 (61.7)	
I should be able to do whatever I want online.				
Disagree	252 (36.1)	161 (39.2)	91 (31.7)	4.083, 1, 0.043
Agree	446 (63.9)	250 (60.8)	196 (68.3)	
The School has no right to interfere in my online activities.				
Disagree	139 (19.9)	80 (19.5)	59 (20.6)	0.127, 1, 0.722
Agree	559 (80.1)	331 (80.5)	228 (79.4)	

Current communication experience and future interaction plans between students and patients are shown in Table 5. Dental students were more open to future communication with patients through SM than medical students (39.7% vs 16.3%; *P* < 0.001). Almost 40% of all students were uncertain about their plans regarding the use of SM for communication with patients when they become doctors.

**Table 5:** Student-patient communication and interaction on social media (SM)

	All students n (%)	Medical n (%)	Dental n (%)	χ2, df, P
When I become a doctor, I’ll use the SM for communication with patients				
Yes	181 (25.9)	67 (16.3)	114 (39.7)	81.686, 2, <0.001
No	249 (35.7)	198 (48.2)	51 (17.8)	
Can’t decide	268 (38.4)	146 (35.5)	122 (42.5)	
How would you react if a patient sends you a friend request/social media tracking?				
I will accept the request	169 (24.2)	50 (12.2)	119 (41.5)	87.344, 4, <0.001
I will decline the request without any further action on my part	97 (13.9)	76 (18.5)	21 (7.3)	
I will decline the request and send a personal message giving the reason for declining	27 (3.9)	17 (4.1)	10 (3.5)	
I will decline the request and discuss my decision with the patient in person during their next visit	137 (19.6)	99 (24.1)	38 (13.2)	
I will not do anything (either accept or decline the request)	268 (38.4)	169 (41.1)	99 (34.5)	
I accepted a patient’s friendship request				
Yes	104 (14.9)	23 (5.6)	81 (28.2)	68.235, 1, <0.001
No	594 (85.1)	388 (94.4)	206 (71.8)	
I sent a friend-request to a patient				
Yes	20 (2.9)	5 (1.2)	15 (5.2)	0.002*
No	678 (97.1)	406 (98.8)	272 (94.8)	

*Fisher exact test.

Dental students significantly more frequently responded that they would accept (41.5% vs 12.2%; *P* < 0.001) and had already accepted (28.2% vs 5.6%; *P* < 0.001) a friend request/follow/track from patients, and even sent friend requests/follow/track to their patients (5.2% vs 1.2%; *P* = 0.002).

As a final part of the questionnaire, students responded they would find guidelines about e-professionalism useful; dental students were significantly more interested in the existence of such guidelines (73.5% vs 81%; *P* = 0.018).

## Discussion

Our study found ubiquitous SM use in the whole sample. It also found that medical and dental medicine students had similar SM habits and similar attitudes toward e-professionalism. These findings were expected as both professions are based on the same ethical and legal foundations. However, the differences between the groups highlight the need for a better understanding of SM behavior of these groups. Such understanding might provide a basis for developing better guidelines and educational interventions for the students from both schools.

Facebook was the most commonly used SM platform (99%), followed by Instagram (81%) and YouTube (72%). The groups significantly differed in YouTube usage (76.9% medical vs 65.2% dental). Facebook is also the most globally used social networking site, and the most commonly used website in the health care student population (66.9%-98.7%, but 98.7% in the dental student population) (15,16,18,25). Instagram and YouTube are catching up fast, with their popularity growing rapidly among teenagers and young adults (26). Instagram is based on fast visual exchange, and because the “older” generation of SM users has not caught on as much as the “younger” users, it is a preferred SM platform for non-professional interaction (27). YouTube content is an integral part of the online curriculum for UZSM students, which can explain the significant difference in usage between the two groups. Suner et al identified watching YouTube videos from the smartphone as a useful tool for educational purposes among dental students (28). Implementation of YouTube in e-learning courses could increase the use among dental students, but with proper guidance (29). Our respondents were heavy SM users, with 93.1% accessing daily and a third accessing more than ten times a day. This is also reported in the literature, amplifying the concern that SM use could be addictive (3,9,30), especially since the current generation of students considers the internet an extension of themselves (31). SM were accessed predominantly by mobile phones (98.3%), with no dental student reporting the use of a desktop computer, which is in accordance with the study by Zupanic et al (32). This is important, as mobile device users are heavier users and interact more. The majority of students (59%) used SM for both private and professional purposes, leaving the possibility of openly mixing professional and private interactions on SM; similar observations were reported by George et al (33). Unclear boundaries of personal-professional interactions have been previously documented as areas of concern for students (34), emphasizing the need to develop students’ professional identities by implementing SM interventions into the medical curricula (35).

Our research showed that students understood the great potential of SM and believed that the benefits of SM use greatly outweigh the risks (59.6%). SM enable students to interact professionally with peers and superiors, access educational content, and engage in positive self-promotion and marketing, all of which has been shown to be able to boost one’s career (36,37). However, after initially stating high professional beliefs, the majority of students showed they were unaware of the consequences of online activity, with just 35.8% acknowledging the effect of the online activity on their professionalism. They also stated (63.9%) that they should be allowed to do what they want online, and that the school did not have the right to interfere with their online activity (80.1%). This clearly demonstrates a “cognitive dissonance,” ie, a discrepancy between what students thought they should do and what they thought they actually did (31,38). Students often engage in unprofessional online behavior (18,39,40). As the concept of e-professionalism is new to the Croatian health care system (both education and practice), there exist no guidelines or widespread awareness of these issues, and students were not informed of these problems. Students' awareness should be increased by developing guidelines and an active implementation of these guidelines within the curriculum (15,16,41,42).

Behaviors and posts on SM that were considered unprofessional by our respondents were in line with previous research (15,17,43). Interestingly, we observed significant differences between medical and dental students. Significantly more medical students believed that posting patient photographs on social media to be unprofessional. This could be a consequence of dentistry being more of a visual, esthetically driven profession. Dental professionals and practices post and share patients' photos and video material on SM as part of marketing activities (“before and after shots”), educational materials, and professional self-promotion. Dental students are also more exposed to visual representations during their studies, which could make them more desensitized to such content (44).

Research has shown that clear violations of professional behavior (confidentiality, falsifying credentials, and inappropriate patient communication) evoked disciplinary action by regulatory agencies such as medical boards (45). Furthermore, 40% of board members indicated that they would investigate even borderline unprofessional behavior, such as posting images of a person drinking alcohol (45). Only around 28% of our respondents found this type of posts to be unprofessional.

The two studied groups significantly differed in the willingness to confront authority on SM. Dental students significantly more frequently perceived that critical comments about faculty (53% vs 39.7%), teaching materials, study program, school, or university (36.2% vs 25.1%) on SM were unprofessional. Previous studies have presented similar results, but these studies, besides medical and dental students, also included students from other health care professions (18,46). Chretien et al (47) listed two main reasons why medical students use SM: to access information and a platform for advocacy, and secondary to take control of their digital footprint, and to achieve a sense of equalization within the medical hierarchy. To the best of our knowledge, this is the first study that directly compared attitudes between medical and dental students. As the UZSDM has a smaller student population than the UZSM, a closer connection with superiors and faculty could explain why more UZSDM students regarded criticism toward superiors as unprofessional.

Student-patient interactions are especially problematic as students are not yet professionals, but should abide by the same ethical and professional rules as graduated doctors of medicine and dental medicine. In traditional relations, a professional separation between doctors and patients is more easily maintained, whereas on SM this border can be vague (48,49). Practicing doctors usually have a more ethical and professional approach to online interaction, while younger generations have less sense of a hierarchy and see the internet as an equalizer that opens doors (50). In our study, dental students were more open to communication through SM than medical students. The overall prevalence of students' unprofessional behavior was low and is in line with findings from other research, where medical students generally felt they should avoid befriending patients (33). Contrary to this, Jafarey et al found that 62% of the students believed it was acceptable to befriend patients (49). However, a significantly higher proportion of dental students who were willing to befriend patients might be explained by dentistry being a business-driven profession. Similarly, in a UK study, dental undergraduate students believed that given that dentistry was a business, different expectations regarding SM use were placed on dental students compared with medical students (51). Parmar et al found that 44% of patients were happy to be contacted by their dentists on SM, while 74% of dentists agreed that friendships with patients on SM were inappropriate. Still, 29% accepted friend requests from their patients (52). As a much larger proportion of dental students in Croatia ends up working in the private sector, the willingness and openness to attract and retain patients could explain this type of an SM interaction. Furthermore, dental students and their patients are interacting in real life, in the dental office of the UZSDM, far longer and more closely than medical students do with their patients. Usually, they have the same patient on each of the specific dental courses through the whole semester, whereas medical students usually interact with patients in a one-time event. This could create more bonding between patients and dental students, possibly easing the choice of befriending the patient.

Students stated they would find guidelines about e-professionalism useful (73.5% medical vs 81% dental students). A decade ago, US medical schools recognized the lack of policies related to SM use (53), but since then, schools have developed specific SM guidelines (54-56). In spite of this, recent findings indicate that students should be included in the development of guidelines (33) and that the guidelines should address befriending patients and having a separate professional profile. There is distinction between disseminating guidelines and formally integrating SM instruction into the medical curricula (43). A most beneficial approach would be to implement a training on appropriate use of SM into the curriculum on e-professionalism. Topics that should be addressed include editing one's online presence, managing friend requests from patients, dealing with colleagues who post harmful content, conducting internet searches on patients, and discussing boundaries to identify potential harms associated with SM use (57). As a result of these study findings, the first guidelines for medical and dental students in Croatian have been published (58) and a new elective subject (within each respective school curriculum) had been developed.

Limitations of this study are volunteer bias and limited sample selection (not all medical and dental schools in Croatia were included). Thus, the results may not be generalizable to all the medical and dental schools in Croatia. We used a convenient sample from a two-center cross-sectional study. Convenient sampling was used deliberately to access more students from the UZSDM to enable comparison with a larger sample size from the UZSM. Second- and fifth-year UZSM students and UZSDM students from all 6 years were asked to participate in the study, because researchers could directly access these students during the subjects they taught. The sample is representative only of the students of these two schools because the study had a large response rate and the sample matched age and gender distribution of the population.

Furthermore, between data collection and paper submission several new SM platforms have gained popularity (Snapchat, TikTok, Instagram Reels). Snapchat as a SM platform that deletes posted content after it was visible for a short period of time by two directly connected users, greatly limits the possibility to disseminate unprofessional behavior to broader audience. Besides, Snapchat and TikTok are mostly used by adolescents, hence they were not of interest to us (59-61). The study was conducted in 2019, and almost three years have passed between data collection and publishing. Our study is susceptible to information and recall bias as it was based on students' self-reporting.

In conclusion, social media use is ubiquitous in the studied population. Both student groups had high awareness of e-professionalism, but dental students were more desensitized to visual representations of patients, and more prone to SM patient interaction, which potentially puts them at a higher risk of unprofessional behavior.

The findings of this study indicate the need for guidelines development and for incorporating e-professionalism subjects into the schools’ curricula. Further studies should expand the range of the investigated SM and assess the effectiveness of guidelines implementation in the curriculum.
