# Supplementary material for: Social media use, habits and attitudes toward e-professionalism among medicine and dental medicine students: a quantitative cross-sectional study
Source: Croat Med J. 2021 Dec;62(6):569–79. doi: 10.3325/cmj.2021.62.569 (PMC8771237; doi:10.3325/cmj.2021.62.569)
Supplement: Supplementary material [file CroatMedJ_62_s004.pdf]

1. What is your gender?

a) male
b) female

2. How old are you?

______

3. What do you study?

a) Medicine, integrated undergraduate and graduate study of medicine
b) Dental medicine, integrated study of dental medicine

3.a. What year are you in?

a) 1
c) 2
d) 3
e) 4
f) 5
g) 6

4. Do you use any of the social networking websites? (e.g. Facebook, Instagram, LinkedIn and the like)?

a) Yes
b) No

5. How familiar are you with the following social media platforms?

	Never heard of it	Heard of it; not sure of its use	Familiar with its use	Very familiar with its use
a) Facebook				
b) Twitter				
c) Linked In				
d) YouTube				
e) Google+				
f) Pinterest				
g) Tumblr				
h) Instagram				

6. How competent are you with using the following social media platforms?

	Not at all competent	Beginner	Competent	Highly competent
a) Facebook				
b) Twitter				
c) Linked In				
d) YouTube				
e) Google+				
f) Pinterest				
g) Tumblr				
h) Instagram				

7. How important are the following reasons for using social media platforms?

	Not at all important	Somewhat important	Very important
a) To keep in touch with current friends and family members			
b) To reconnect with old friends whom I have lost touch with			
c) To connect with people who share my hobby			
d) To communicate with peers and teachers about educational issues			

8. How important are the following reasons for not using social media platforms?

	Not at all important	Somewhat important	Very important
a) Lack of knowledge			
b) Lack of time			
c) Lack of interest			
d) Lack of any perceived value			
e) Concern about harm that social media might do to my professional image			

9. How often do you check on your online presence (e.g. Google yourself)?

a) Never
b) Occasionally (usually not more than once a month)
c) Regularly (at least once a week)
d) Frequently (daily)
e) Very frequently (several times a day)

10. How often do you search for your photos on Google Image?

a) Never
b) Occasionally (usually not more than once a month)
c) Regularly (at least once a week)
d) Frequently (daily)
e) Very frequently (several times a day)

11. How your social media profile is registered:

	a) Under my real name	b) Under a different name	c) Have two profiles, one under my real name and another under a different one	d) Have more than two profiles	e) Don't have a profile
Facebook					
Instagram					
Pinterest					
Tumblr					
Twitter					
LinkedIn					
YouTube					
Google+					

12. How important are the following reasons for checking on your online presence?

	Not at all important	Somewhat important	Very important
a) To make sure that the information posted are true			
b) To make sure that the information posted are complete			
c) To make sure that the information posted are professional			

13. What action have you taken if you find information that you believe should never be posted?

Check all answers that apply.

a) I deleted people from my friends list
b) I deleted all comments made on my profile
c) I removed my name from photo name tags
d) I have taken no action

14. Have you ever found that information about you shared on social media are:

Check all answers that apply.

a) Inaccurate
b) Incomplete
c) Unprofessional
d) Other (please specify) ________________________

15. What do you use social media most often for?

a) Exclusively for personal purposes
b) For both personal and professional purposes
c) Exclusively for professional purposes

16. Please answer the following Yes / No questions on your social media usage (Facebook, Instagram, LinkedIn etc.):

	Yes	No
a) I have accepted a friend request from a patient		
b) I have sent a friend request to a patient		
c) I have accepted a friend request from a teacher		
d) I have sent a friend request to a teacher		
e) I have googled a teacher		

17. What concerns do you have about social media use?

	No concerns at all	Some concerns	Strong concerns
a) Public perceptions of unprofessional behaviour by me.			
b) Family perceptions of unprofessional behaviour by me.			
c) Public perception of unprofessional behaviour by my peers.			
d) Public perceptions of my school (School of Medicine/School of Dental Medicine).			
e) Public perceptions of my profession.			
f) Violations of patient confidentiality.			
g) Posting of inaccurate medical information accessible to patients.			

18. To what extent do you agree with the following statements?

	Strongly disagree	Disagree	Agree	Strongly agree
a) One of the responsibilities of a teacher is to counsel students on the appropriate use of social media.				
b) Patients use social media to get medical / dental information.				
c) The benefits of social media overweigh the risks of their use.				
d) As a medicine / dental medicine graduate, it is my obligation to keep abreast with the current trends in the use of social media.				
e) Guiding patients to online information is a new responsibility of physicians in the digital age.				

19. Your personal data can be accessible on social media sites without your explicit intention or consent?

a) True
b) False

20. The School has developed guidelines for social media use?

a) True
b) False

21. When I become a physician I will use social media in my interaction with patients

a) Yes
b) No
c) Can't decide on information given

22. How will you react if a patient sends you a friend request / request for monitoring on social media?

Choose a response from the list of answers provided below:

a) I will accept the request
b) I will decline the request without any further action on my part
c) I will decline the request and send a personal message giving the reason for declining
d) I will decline the request and discuss my declination with the patient in person during his/her next visit
e) I will not do anything (either accept or decline the request)

23. How will you react if a teacher sends you a a friend request / request for monitoring on social media?

Choose a response from the list of answers provided below:

a) I will accept the request
b) I will decline the request without any further action on my part
c) I will decline the request and send a personal message giving the reason for declining
d) I will decline the request and discuss my declination with the teacher in person next time we meet
e) I will not do anything (either accept or decline the request)

24. How often do you check on your social media accounts?

a) More than 10 times a day
b) 5 – 10 times a day
c) 2 – 4 times a day
d) Once a day
e) 2 – 3 times a week
f) Once a week or less

25. What device do you mostly use to access social media?

a) Desktop computer
b) Laptop computer
c) Mobile device (tablet/mobile phone)

26. How selective are you about accepting a friend request / request for monitoring on social media (ONLY ONE RESPONSE for each social media platform listed below)?

	Facebook	Instagram	LinkedIn	Twitter
a) I accept friend requests only from people I know well and family members.				
b) I accept friend requests from people I have already met in person several times.				
c) I accept friend requests from people I have met in person only once.				
d) I will accept friend requests if I recognise the names or if we have some mutual friends although we have never met in person.				
e) I accept friend requests from people I have never met in person and whom I don't know.				
f) I don't use this social media platform.				

27. Have you ever changed basic privacy settings on social media platforms?

a) Yes
b) No
c) I don't know

28. Please check all the reasons why you have changed your privacy settings on social media:

a) Protection of my personal information from strangers
b) Risk that my profile may be seen by my current or future employer
c) I held a position that required me to act as a role model for others (mentor, teacher, demonstrator and the like)
d) Advice from my peers and friends
e) I don't trust privacy setting safety on social media
f) Other (please specify) ____

29. Is your personal profile on social media:

a) Completely publicly available (anyone can view the complete content of your profile)
b) Of limited public availability (strangers/people outside of your circle of acquaintances can view some information but not your complete profile content)
c) Completely private (only people whom you accepted as “friends” /or those to whom you are connected can view your profile)
d) I don't know

30. Current literature suggests that professional behaviour may depend on the context, environment and surroundings in which it occurs. In what situations do you believe medical / dental medicine students should ALWAYS behave professionally?

(Check all the answers that you believe apply)

a) In the school (e.g. classrooms, training facilities)
b) At the clinic (outpatient clinics, hospitals)
c) In public in their free time

31. Review the following list of potential negative outcomes resulting from unprofessional online behaviour and check to what extent do you agree or disagree.

(Scale: *1 – Strongly disagree; 2 – Disagree; 3 – Neither agree nor disagree; 4 – Agree; 5 – Strongly agree*)

a) People can make wrong assumptions about you based solely on the content of your post.
b) There is a possibility that your online behaviour might have an impact on perception of others in your profession.
c) It is possible that your potential employer will not hire you or invite you for an interview due to information about you found online.
d) You may lose a position you already hold (as an employee or student) due to information about you found online.
e) Sharing privileged patient information on social media without their consent is deemed to be inadmissible.

32. How would you describe your usual activities or behaviour on social media platforms in reference to the following definitions?

Active behaviour: commenting, posting links or photos, sending invitations / friend requests

Passive behaviour: reading, viewing your photos or other contents related to you but posted by others

a) More active than passive
b) Half active half passive, approximately
c) More passive than active
d) I don't have a social media account

33. Taking into consideration your answer to the previous question what strategy do you believe provides better protection of your online image?

a) Abstaining totally from social media and not having an account at all.
b) Maintaining account to keep track of posts that may include information about you (photos and the like)

34. To what extent do you agree with the following statements about professionalism in healthcare?

	Strongly disagree	Disagree	Agree	Strongly agree
a) I know well what constitutes professional behaviour and what is expected of me as a current/future professional.				
b) I strongly agree with expectations for professional behaviour and make a conscious effort to comply with them in every aspect of my life.				
c) High-level professional behaviour should also be expected of students from the very beginning of their studies.				
d) A little leniency should be shown if unprofessional behaviour occurs in the first years of professional education.				

35. To what extent do you agree with the statements about professionalism standards related to online activities?

	Strongly disagree	Disagree	Agree	Strongly agree
a) Professionalism in online activities is as important as in traditional (offline) environments.				
b) It is not always possible to maintain professionalism in online activities.				
c) People have the opportunity to post photos and document aspects of their professional life which would otherwise remain private.				
d) Social media have removed protection of professionals against the public.				
e) Professionals cannot actually fully relax.				
f) The risks of social networking software greatly overweigh the benefits.				
g) Healthcare professionals should be restricted from using social networking software due to too much of a risk.				
h) Healthcare professionals should be banned from using social networking software due to too much of a risk.				
i) I believe that my online activities do not affect me as a professional.				
j) I should be able to do whatever I want online.				
k) The School has no right to interfere in my online activities.				

36. To what extent do you agree with the following statements?

(Scale: *1 – Strongly disagree; 2 – Disagree; 3 – Agree; 4 – Strongly agree*)

a) I believe discussion on online professionalism to be more important for my profession than for any other.
b) I believe discussion on online professionalism to be more important for my healthcare profession (physicians of different specialties) in respect to any other profession.

37. Which of the following types of posts / documents (posted online) do you believe to be unprofessional?

a) Image of a person drinking alcohol.
b) Image of a person who looks undeniably drunk.
c) Posting a status update to describe considerable alcohol consumption at a party.
d) Posts that show illicit drug use.
e) Posts that display patient information.
f) Photos of patients.
g) Posts that describe interaction with a patient, not revealing any personally identifiable information.
h) Swearing or inappropriate language.
i) Obscene gestures in photos (the middle finger and the like).
j) Misdemeanours.
k) Advertising of pharmaceutical or health products without disclosing any conflict of interest.
l) Posts that include overtly sexual content.
m) Posts that contain partial nudity.
n) Display of the current love status.
o) Display of the membership of certain online groups dealing with controversial issues.
p) Stating opinion in comments on controversial issues.
q) Critical comments on faculty.
r) Critical comments on teaching materials, study programme, school or university.
s) Expressing attitudes of superiority (based on professional status).

38. Do you think that guidelines for professional use of social media would be useful to other physicians?

a) Yes
b) No

39. Have you ever posted or commented something online that you have later come to regret?

a) Yes
b) No

If yes, please explain: ____

40. Has anyone else ever posted things about you online that made you feel embarrassed or look unprofessional?

a) Yes
b) No

If yes, please explain: ____

41. Have you ever noticed things posted online by your peer that you considered unprofessional?

a) Yes
b) No

If yes, please explain: ____

42. Do you think that guidelines for professional use of social media would be useful to you?

a) Yes
b) No

43. To what extent do you agree with the following statements?

	Strongly disagree	Disagree	Neither agree nor disagree	Agree	Strongly agree	Don't know
a) It is ethically acceptable for a physician to visit patient social media profile.						
b) It is ethically acceptable for a physician to communicate (e.g. share personal messages) with a patient through personal social media account for easier social interaction.						
c) It is ethically acceptable for a physician to communicate with a patient through social media as part of his/her care for patients and the patient healthcare process.						
d) Social media have the potential to improve communication between a physician and a patient.						
e) Communication with a patient through social media can be achieved without compromising physician-patient confidentiality.						
